# Metformin induces ER stress-dependent apoptosis through miR-708-5p/NNAT pathway in prostate cancer

**DOI:** 10.1038/oncsis.2015.18

**Published:** 2015-06-15

**Authors:** J Yang, J Wei, Y Wu, Z Wang, Y Guo, P Lee, X Li

**Affiliations:** 1 Department of Basic Science and Craniofacial Biology, New York University College of Dentistry, New York, NY, USA; 2 Tongji Hospital, Wuhan, China; 3 Department of Urology, New York University, Langone Medical Center, New York, NY, USA; 4 Department of Pathology, New York University, Langone Medical Center, New York, NY, USA

## Abstract

Although the antitumor role of metformin has been widely reported, the molecular mechanism of this biguanide agent in the inhibition of tumor progression remains unclear. Here, we identified miR-708-5p as a novel target of metformin in prostate cancer cells. Metformin promotes increased expression of miR-708-5p, leading to suppression of endoplasmic reticulum (ER) membrane protein neuronatin (NNAT) expression and subsequently induces apoptosis of prostate cancer cells through the ER stress pathway. Further, miR-708-5p-induced knockdown of NNAT is associated with downregulated intracellular calcium levels and induced malformation of ER-ribosome structure revealed by electronic microscopy. Meanwhile, the unfolded protein response regulator CHOP, p-eIF2α, calreticulin, GRP78 and ATP2A1, all of which are also considered as ER stress markers, are upregulated by metformin and miR-708-5p. Taken together, our findings clearly demonstrate that metformin stimulates increased expression of miR-708-5p to target the NNAT-mediated response to ER stress and apoptosis. This novel regulatory mechanism of metformin in prostate cancer cells not only advances our knowledge on the molecular mechanism of metformin but also provides a promising therapeutic strategy by targeting miR-708-5p and NNAT for prostate cancer treatment.

## Introduction

Metformin has been used as a first-line antidiabetes drug for decades. Accumulating epidemiology reports show that metformin, but not other oral hypoglycemics, decreases the risk of developing prostate cancer.^1^ As a biguanide reagent, metformin enters the mitochondria and reduces oxidative phosphorylation, which causes decreased cellular ATP, leading to an antiproliferative effect through AMPK pathway.^2^ Meanwhile, through intervening metabolic pathways, metformin is also considered an ideal agent to be used in adjuvant and advanced disease trials for cancer therapy.^3^ Increasing evidence has shown that biguanides impede nucleotide synthesis^;^^4^ however, how metformin regulates microRNA (miRNA) expression profiles in prostate cancer has not been thoroughly investigated.

MiRNAs are a group of noncoding RNAs that regulate gene expression. In normal cells, miRNAs are responsible for maintaining homeostasis across a number of cellular processes, yet in transformed cells, miRNA dysregulation can promote cancer progression.^5^ miRNAs target fundamental genes that regulate proliferation, apoptosis and migration, and can be used as diagnostic markers for prostate cancer.^6^ Reports also showed that miRNAs targeting cell growth and survival pathways can be critical in the mediation of cell transformation.^7^ Furthermore, miRNAs are also involved in remolding tumor microenvironment in cancer progression^8, 9^ and have enormous potential for cancer therapeutics.^9^ Specifically, miR-708-5p was found to have a metastasis-suppressive role by targeting endoplasmic reticulum (ER) protein neuronatin (NNAT), and by downregulating intracellular calcium levels in breast cancer. Moreover, low expression level of miR-708-5p in the lymph node and distal metastases indicates a therapeutic potential of this small noncoding RNA.^10^

NNAT has important roles in both physiological and pathological processes. It was initially identified as a signal-transduction factor with possible involvement in terminal brain differentiation^11^ and neuronal growth and differentiation.^12^ A recent report shows that NNATβ has a critical role in the pathogenesis of type 2 diabetes by mediating high glucose-induced apoptosis of pancreatic β-cells.^13^ As mentioned earlier, NNAT has been reported to stimulate tumor metastasis in breast cancer.^10^ Epigenetic analysis also shows that hypermethylation of NNAT is associated with the tumorigenesis of pediatric acute leukemia.^14^ Because of its association with increasing cell proliferation and lowering patient survival, NNAT is suggested to be used as a prognostic biomarker for glioblastoma multiforme.^15^ These clinical findings on NNAT suggest that it may be a therapeutic target for inhibiting cancer progression in multiple types of malignancies. Interestingly, NNAT was found to mediate calcium signal and its ectopic expression results in increased intracellular calcium level and ER stress associated with neuropathy.^16^

As the intracellular calcium level and ER stress are important to maintain normal cellular function in general, we asked whether NNAT regulates prostate tumor cell growth through its regulation of intracellular calcium level and ER stress, and whether metformin targets NNAT to inhibit prostate cancer growth. We performed serial experiments to address these questions: first, through a microRNA array screening, we identified the miRNA expression profile of metformin-treated prostate cancer cells. Second, we confirmed that miR-708-5p, which has the largest fold change under the treatment of metformin, targeted NNAT. Third, miR-708-5p exhibited an ER stress-dependent proapoptotic function mediated through NNAT in prostate cancer cells. Taken together, our findings reveal a novel miRNA regulatory role of metformin in inhibition of prostate cancer and a new antitumor mechanism of metformin by targeting miR-708-5p/NNAT pathway to stimulate prostate cancer cell apoptosis.

## Results

### Metformin regulates the miRNA expression profile and upregulates miR-708-5p in prostate cancer cells

**Figure 1:**
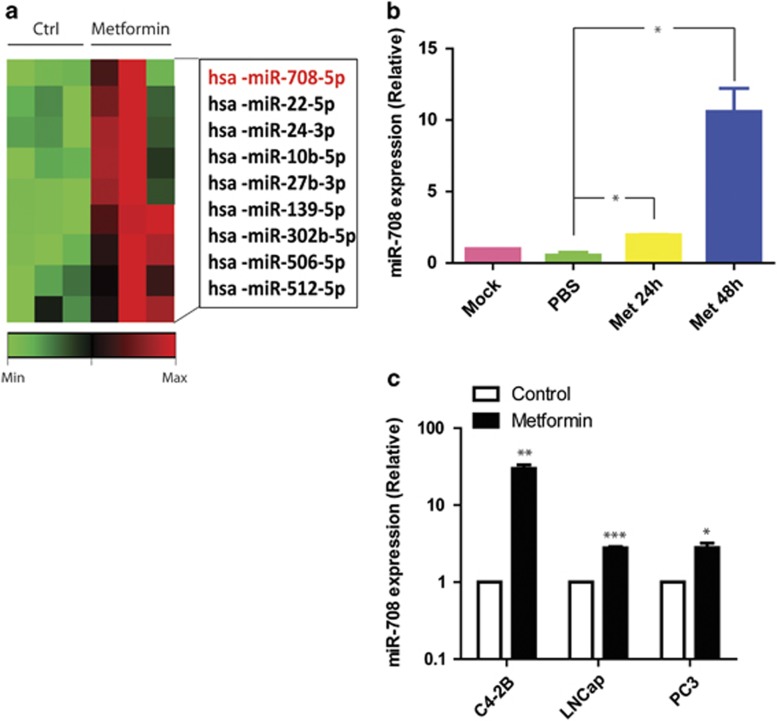
Metformin increases miR-708-5p expression in prostate cancer cells. (**a**) MicroRNA array was performed with total RNA from C4-2B cells (*n*=3) treated with or without metformin (final concentration=5 mm) for 48 h. (**b**) Quantitative real-time-PCR (qRT-PCR) analysis of miR-708-5p expression in C4-2B cells. C4-2b cells were treated with 5 mm metformin for 24 or 48 h. Non-treated (mock) and phosphate-buffered saline (PBS)-treated cells were used as controls. (**c**) qRT-PCR analysis of miR-708-5p expression in C4-2B, LNCaP and PC3 cells, which were treated with 5 mm metformin for 48 h.

To understand the function of metformin in regulating the miRNA expression profile in prostate cancer cells, we performed miRNA array assay with total RNA of metformin-treated or -nontreated (control) C4-2B cells (*n*=3). The heatmap diagram shows the results of 374 human miRNAs for each sample; red represents an increasing expression and green represents a decreasing expression as illustrated by the color bar at the bottom (data have been uploaded to Figshare). Among all the miRNAs, miR-708-5p shows the greatest magnitude change (Figure 1a and Supplementary Figure S1). Further detection of the miR-708-5p expression levels was performed with C4-2B cells treated with metformin for 24 and 48 h, using non-treated cells (mock) and phosphate-buffered saline-treated cells as controls. The expression of miR-708-5p increased significantly after 24 h metformin treatment and continued to increase strongly after longer treatment of 48 h (Figure 1b). MiR-708-5p expression level in other metformin-treated and -non-treated prostate cell lines such as LNCaP and PC3 were also determined by quantitative PCR. Expression of miR-708-5p in all three cell lines showed the same trend after metformin treatment (Figure 1c).

### miR-708-5p inhibits the expression of NNAT and the uptake of calcium by ER

**Figure 2:**
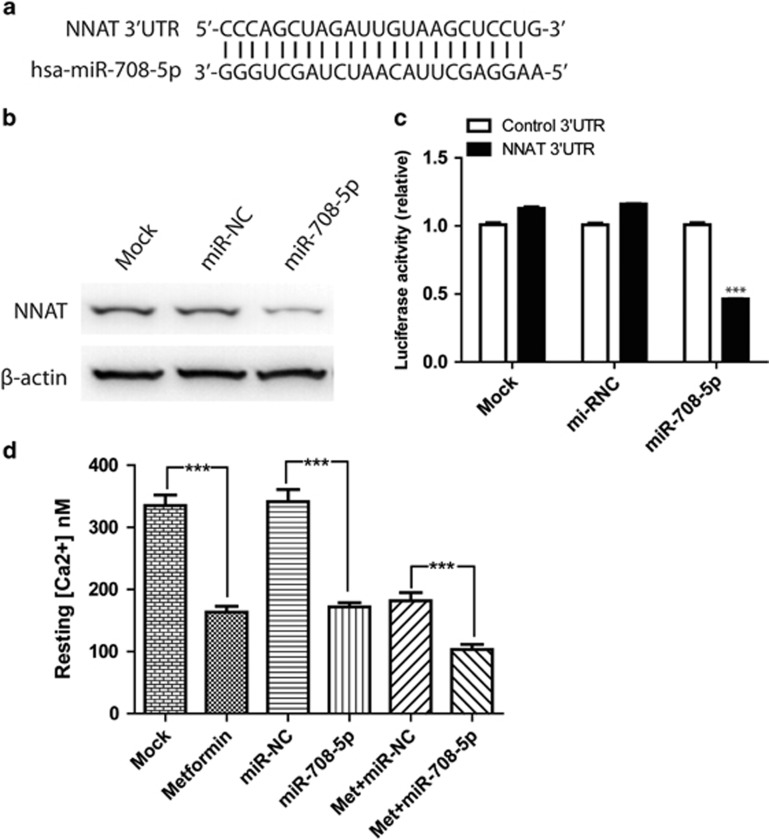
MiR-708-5p inhibits NNAT expression by targeting 3′-UTR. (**a**) Sequences of miR-708-5p and the potential miR-708-5p binding site at the 3′-UTR of NNAT. (**b**) Western blot analysis of endogenous NNAT in C4-2B cells (ock), and C4-2B cells treated with miR-NC or miR-708 for 48 h. β-Actin serves as an internal control. (**c**) NNAT 3′-UTR construct or the control construct were co-transfected into C4-2B cells along with miR-708-5p or miR-NC and assayed for luciferase activity. Firefly luciferase values were normalized to *Renilla* luciferase activity. (**d**) Respective resting intracellular [Ca^2+^] of mock, metformin, miR-708-5p mimic, control miRNA mimic, metformin+miR-708-5p mimic or metformin+control miRNA mimic-treated C4-2B cells were determined by Fura-2 indicator.

Based on bioinformatic analysis, the sequence of mature hsa-miR-708-5p (23 nt) matches the 3′-untranslated region (UTR) of NNAT mRNA (Figure 2a). To test the effect of miR-708-5p on NNAT expression in prostate cancer cells, we transfected either the synthesized miR-708-5p or the negative control mimic (miR-NC) into C4-2B cells and performed western blots to detect NNAT expression level. The result showed that miR-708-5p significantly suppressed the expression of NNAT in prostate cancer cells (Figure 2b). Meanwhile, metformin showed similar effect in inhibiting NNAT expression (Supplementary Figure S2). To further confirm if miR-708-5p targets the 3′-UTR of NNAT, a dual-luciferase reporter vector, which harbors the 3′-UTR sequence of NNAT, was constructed. We performed a co-transfection of the reporter plasmid with either the miR-708-5p or miR-NC into C4-2B cells. Subsequently, a dual-luciferase (firefly luciferase and *Renilla* luciferase) activity assay with the reporter plasmid included as mock control was conducted. MiR-708-5p showed a significant suppressive effect on the firefly luciferase activity compared with miR-NC and mock control (Figure 2c). Because NNAT expression is responsible for the uptake of calcium by the ER, we tested if miR-708-5p- and metformin-induced knockdown of NNAT changed the level of calcium in the cytoplasm and the reuptake to ER. The intracellular calcium level was evaluated in C4-2B cells, which were treated with metformin, miR-708-5p mimic or both. As expected, miR-708-5p mimic alone causes aberrant low level of intracellular calcium. Interestingly, metformin also reduces the intracellular calcium level, which has not been reported. This indicates that miR-708-5p could mediate metformin's actions in tumor cells. Notably, when cells were treated with both, the intracellular calcium levels were downregulated even further (Figure 2d).

### Metformin and miR-708-5p induce the ER stress of prostate cancer cells by targeting NNAT

**Figure 3:**
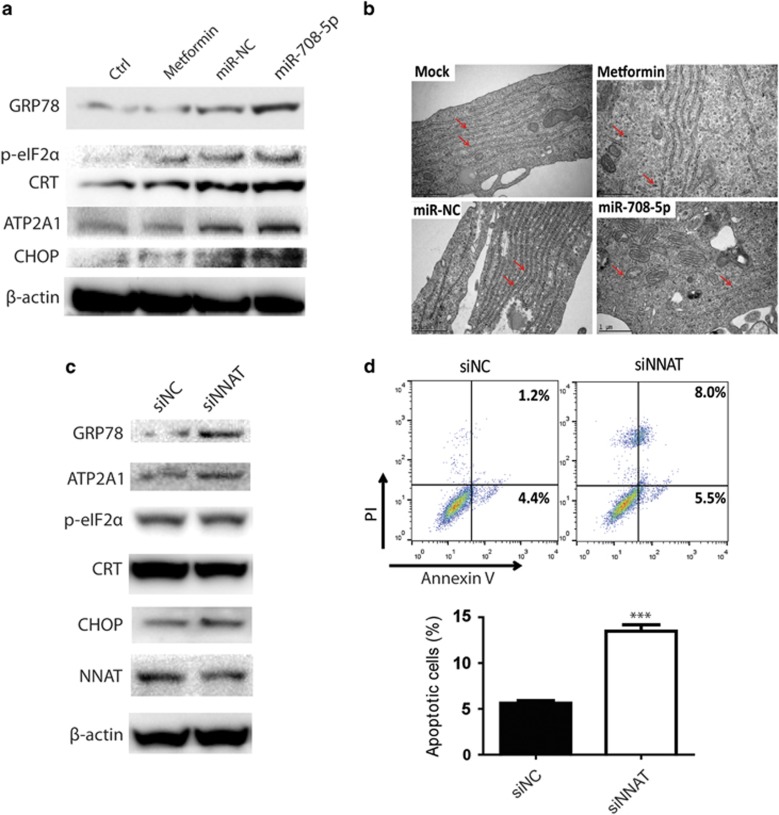
Metformin and miR-708-5p induce ER stress through inhibition of NNAT expression. ER stress-related markers were measured by western blot after lysis of C4-2B cells. C4-2B cells were treated with (**a**) miR-708-5p (final concentration=100 nm), metformin (final concentration=5 mm) or (**c**) NNAT siRNA (final concentration=20 nm) for 48 h. (**b**) The ER structure of C4-2B cells were observed with Phillips CM12 transmission electron microscope. (**d**) Annexin V/PI staining and flow cytometry analysis of C4-2B cells were transfected with NNAT siRNA for 48 h.

The localization of NNAT in ER was reported to be important in maintaining the homeostasis of intracellular calcium levels, whereas abnormal calcium levels in the ER could cause ER stress and apoptosis of cells.^16^ To investigate the function of miR-708-5p in the process of ER stress, a series of ER stress markers, GRP78, p-eIF2α, ATP2A1, calreticulin (CRT) and CHOP, were tested by western blotting. Metformin upregulated p-eIF2α, CRT and CHOP, whereas miR-708-5p transfection increased the levels of GRP78, p-eIF2α, ATP2A1 and CHOP, indicating that both metformin and miR-708-5p can cause ER stress in prostate cancer cells (Figure 3a). Furthermore, to confirm the impact of metformin and miR-708-5p on the ER, we viewed the subcellular structure of C4-2B cells, which were treated with metformin or transfected with miR-708-5p. Consistent with the upregulation of ER stress markers, the malformation and detachment with ribosome of the ER was also observed under the treatment of metformin and transfection of miR-708-5p (Figure 3b). To understand whether metformin and miR-708-5p induces ER stress and apoptosis through NNAT pathway, the endogenous NNAT expression level of C4-2B cells was deleted by NNAT small interfering RNA (siRNA). By testing the ER stress markers, it was found that there were significant increases in the expression levels of GRP78, ATP2A1 and CHOP following the downregulation of NNAT by siRNA (Figure 3c). Annexin V/PI staining and flow cytometry analysis showed that NNAT siRNA can also induce apoptosis in C4-2B cells (Figure 3d). These results imply that metformin and miR-708-5p induce apoptosis and ER stress in prostate cancer cells by targeting NNAT.

### Metformin and miR-708-5p induce apoptosis of prostate cancer via ER stress

**Figure 4:**
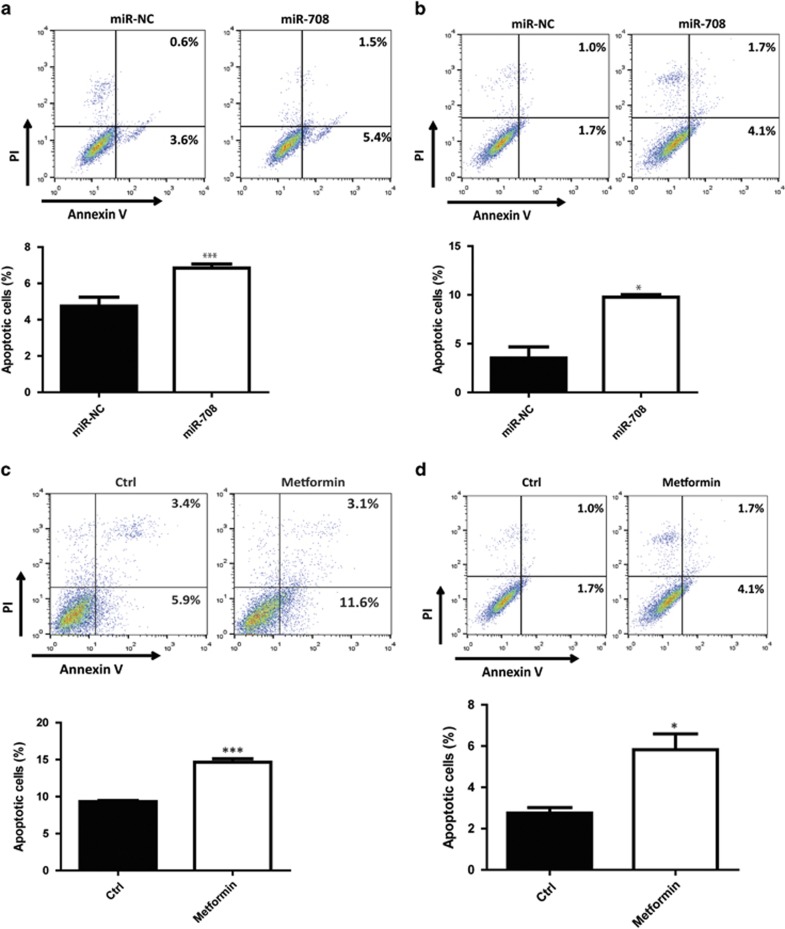
Metformin and miR-708-5p induce apoptosis of prostate cancer cells. Induction of apoptosis of (**a**) C4-2B and (**b**) LNCaP cells by miR-708-5p mimic (final concentration=100 nm) for 48 h was measured by Annexin V/PI staining and flow cytometry analysis. (**c**) C4-2B and (**d**) LNCaP cells were treated with 5 mm metformin for 48 h, followed by Annexin V/PI staining and flow cytometry analysis.

ER stress has been considered as a major cause of cell death in many biological processes. As metformin and miR-708-5p can promote ER stress in prostate cancer cells, they may also function as inducing factors of cell apoptosis. To verify this hypothesis, C4-2B and LNCaP cells were transfected with miR-708-5p mimic or miR-NC for 48 h, followed by Annexin V/PI double staining. Notably, flow cytometry analysis showed that miR-708-5p promotes the population of Annexin V-positive cells, which are considered as apoptotic cells (Figures 4a and b). As an antidiabetes reagent, metformin suppresses cell growth by targeting some key molecules in mitochondria to function as a metabolism-repressive factor. Here, we demonstrated that metformin also promotes the expression of miR-708-5p in prostate cancer. To further explore the function of metformin in the process of cell apoptosis, C4-2B and LNCaP cells were treated with metformin for 48 h and the apoptotic cells were tested by Annexin V/PI double staining. In agreement with the result of miR-708-5p, metformin also increases the apoptotic population of C4-2B and LNCaP cells (Figures 4c and d). Furthermore, PC3, which is a more aggressive prostate cancer cell line with high endogenous NNAT expression level, showed resistance to metformin-induced apoptosis at the same concentration (5 mm) (Supplementary Figure S3). This result implies a protective potential of NNAT from metformin-induced apoptosis. To understand if a timeline existed in the events from metformin treatment to apoptosis induction, C4-2B cell lysate was extracted after metformin treatment for 24 h. Western blotting showed that CHOP expression was markedly increased, indicating that the induction of ER stress started as early as 24 h treatment with metformin, which could preclude to apoptosis (Supplementary Figure S4).

**Figure 5:**
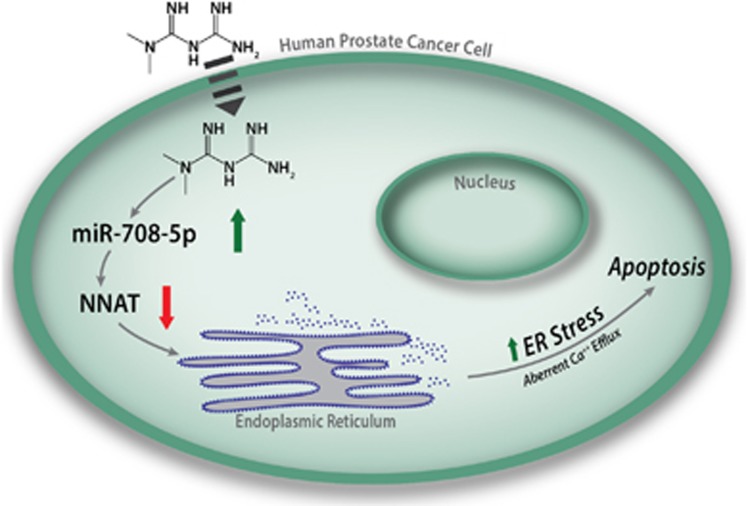
A model demonstrating how metformin induces ER stress and apoptosis of prostate cancer cells via miR-708-5p downregulation of NNAT.

Combined, results from our experiments suggest a novel mechanism by which metformin promotes ER stress in prostate cancer cells, leading to apoptosis as depicted in Figure 5. Notably, metformin inhibits the expression of NNAT by increasing miR-708-5p expression levels. Downregulated NNAT, in turn, attenuates Ca^2+^ efflux from the ER to the cytoplasm and subsequently induces ER stress, as evidenced by ER stress markers and ribosome detachment, which eventually induces the apoptosis of prostate cancer cells.

## Discussion

Metformin's association with decreased risk of prostate cancer has been reported in multiple clinical studies.^17, 18, 19^ Studies also indicate that it may be a promising chemotherapeutic alternative for metastatic castration-resistant prostate cancer.^20^ Our previous study demonstrated that metformin suppressed prostate cancer growth *in vitro* and c-Myc-driven prostate epithelial malignant transformation *in vivo*.^21^ Further investigation of metformin's molecular mechanism and targets will reveal its potential application as a monotherapy or part of a polytherapy in cancer prevention. In the present study, we performed microRNA superarray analysis and identified miR-708-5p as a strong downstream effector of metformin action in C4-2B prostate cancer cells. The regulation of miR-708-5p by metformin has not been reported in any previous studies. Using quantitative real-time-PCR, we confirmed that miR-708-5p was significantly upregulated by metformin in other cell lines including LNCaP and PC3 cells.

MiR-708-5p has a tumor-suppressive role by targeting zinc-finger E-box-binding homeobox 2 as well as polycomb ring-finger oncogene (BMI1) to induce apoptosis and inhibit tumorigenesis in renal cancer.^22^ Furthermore, miR-708-5p also inhibits prostate cancer-initiating cells by targeting CD44 as well as thymoma viral proto-oncogene 2 (AKT2).^23^ Our finding of metformin's regulatory role on miR-708-5p supports the reports that metformin can suppress cancer stem cell's self-renewal.^24^ In this study, we found that NNAT was inhibited by miR-708-5p in prostate cancer cells. Our finding is consistent with a recent study where NNAT was identified as a target of miR-708-5p in breast cancer.^10^ By establishing a dual-luciferase reporter vector, we found that miR-708-5p directly inhibits NNAT expression by targeting the 3′-UTR of NNAT mRNA in prostate cancer cells. Besides its role in facilitating cancer cell metastasis,^10^ we explored and revealed a new function of NNAT in regulating apoptosis in prostate cancer cells. Considering NNAT controls calcium signaling and ER response in neuronal cells,^16, 25^ it was of interest to know whether it regulates these signaling pathways in malignant cells, in particular prostate cancer cells. Our detection of the reduced intracellular calcium levels in C4-2B cells treated with metformin, miR-708-5p mimic or the combinations of both proved a direct connection among Ca^2+^ regulation events in prostate cancer cells. These results indicate that metformin could induce aberrant calcium signaling through miR-708-5p/NNAT pathway. Furthermore, synergetic effect of miR-708-5p mimic and metformin on the contribution of intracellular calcium implies that combination of these two agents may achieve a more favorable chemopreventive effect. The inhibitive potential of metformin on extracellular calcium signaling might be important for the additional effect of combined treatment by metformin and miR-708-5p mimic.^26^ In addition, metformin was reported to have oncogenic target other than miR-708-5p in prostate cancer cells such as c-Myc^21^ and AR,^27^ which could also contribute to the induction of apoptosis. Therefore, combination of metformin and miR-708-5p might enhance each other's effect of antiumor and improve prognosis.

ER homeostasis is regulated by multiple signaling pathways and is responsible for cell survival,^28^ angiogenesis^29^ and apoptosis.^30^ ER stress activates pathways that lead to unfolded protein response, which subsequently has a critical role in stimulating cell apoptosis.^31, 32^ Here, we found that both metformin and miR-708-5p can upregulate unfolded protein response proteins CHOP, p-eIF2α, CRT, GRP78 and ATP2A1, which indicates that metformin stimulates miR-708-5p to induce ER stress in prostate cancer cells. Interestingly, metformin, miR-708-5p and NNAT siRNA can independently induce upregulation of GRP78 and CHOP, which implies that all of them are capable of causing unfolded protein response and ER stress. However, different from NNAT siRNA, metformin and miR-708-5p mimic bypass the ATP2a-CRT pathway. This may be due to additional targets of metformin and miR-708-5p distinct from NNAT. As demonstrated by the electronic microscopy images, ribosome deattachment to the rough ER membrane was significantly increased by metformin and miR-708-5p. Meanwhile, distorted alignment of the ER structure was noticed in prostate cancer cells treated with metformin and/or miR-708-5p mimic. Similar observations in prostate cancer cells transfected with NNAT siRNA further confirmed that metformin could lead to ER stress in prostate cancer cells through an miR-708-p/NNAT-regulated pathway.

ER stress-dependent apoptosis has been recently reported as a promising therapeutic pathway to target to induce cancer cell death.^33^ In this study, we functionally evaluated metformin- and miR-708-5p-induced apoptosis in prostate cancer cells using Annexin V/PI double staining and flow cytometry analysis. As expected, metformin, as well as miR-708-5p, induced apoptosis in prostate cancer cells. Thus, in addition to stimulation of AMPK^34, 35^ and inhibition of mTOR^35, 36^ pathways, metformin can induce cancer cell death by its regulation on calcium signaling and ER response. In summary, we revealed a novel signaling pathway responsible for metformin-induced apoptosis through miR-708-5p targeted NNAT in prostate cancer cells. Our findings illuminated an alternative pathway activated by metformin, which is worth future exploration on its potential application as a therapeutic target for prostate cancer.

## Materials and methods

### Cell lines and cell culture

Prostate cancer cell lines (PC3, C4-2B and LNCaP) were grown at 37 °C in a 5% (v/v) CO_2_ growth chamber. All cell lines were cultured in RMPI-1640 supplemented with 10% fetal bovine serum, 100 U/ml penicillin and 100 μg/ml streptomycin solution. Cell culture media was obtained from Invitrogen (Carlsbad, CA, USA).

### miRNA transfection

Cells were plated in growth medium without antibiotics ~24 h before transfection. Transient transfection of miR-708-5p (Life Technologies-Ambion, Grand Island, NY, USA) or miR-NC (Life Technologies-Ambion) were carried out by using Lipofectamine 2000 (Invitrogen) according to the manufacturer's protocol.

### Quantitative real-time-PCR

miRNA was isolated using miRNeasy Mini Kit (Qiagen, Valencia, CA, USA) following the manufacturer's instruction. Mature miRNAs were assayed by using the TaqMan MicroRNA Assays and Gene Expression Assays, respectively, in accordance with the manufacturer's instruction (Life Technologies-Applied Biosystems, Grand Island, NY, USA). Samples were normalized to RNU6B Endogenous Control (Applied Biosystems), as indicated. The comparative Ct (threshold cycle) method was used to calculate the relative changes in gene expression on the Bio-Rad CFX384 Real-Time PCR System (Bio-Rad, Herclues, CA, USA).

### Apoptosis assays

Apoptosis was assessed by measuring membrane redistribution of phosphatidylserine using an Annexin V-FITC Apoptosis Detection Kit (BD Pharmingen, San Diego, CA, USA). Per-protocol kit instructions, cells were collected, washed two times with phosphate-buffered saline and resuspended in 500 μl of staining solution containing FITC-conjugated annexin V antibody and propidium iodide. After incubation on ice for 30 min, cells were analyzed by flow cytometry. Basal apoptosis and necrosis were identically determined on untreated cells.

### Luciferase assay

The NNAT 3′-UTR reporter construct (catalog number: HmiT011847-MT01) was obtained from GeneCopoeia Inc. (Rockville, MD, USA) along with control construct (catalog number: CmiT000001-MT01). The NNAT construct or control construct was co-transfected into C4-2B cells along with miR-708-5p or miR-NC using Lipofectamine 2000. Forty-eight hours after transfection, firefly and *Renilla* luciferase activities were measured by using the Dual Luciferase Reporter Assay System (Promega, Madison, WI, USA) according to the manufacturer's protocol.

### Intracellular calcium measurements

A well-established method was performed as described previously.^37^ Briefly, metformin or miRNA mimic-treated cells were suspended in HBSS buffer at 10^6^ cells per ml and seeded in 96-well plate at 100 μl per well. After incubating at 37 °C for 5 min, Fura-2 was added at a final concentration of 10 μm and incubated at 37 °C for 30 min. The cells were washed with HBSS (containing 0.2% fetal bovine serum), resuspended at 5 × 10^5^ cells per ml, followed by an incubation at 37 °C for 20 min. The optical density values at 340 and 380 nm were obtained to determine the calcium concentration with the formula: [Ca^2+^]=*K*_d_ × ((*R−R*_min_)/(*R*_max_*−R*)). *K*_d_ was determined by Calibration Kit (Invitrogen; F-6774), *R*=(OD340*−*Blank)/(OD380*−*Blank), *R*_min_=OD340/380 in the presence of 410 mm EGTA (pH=8), *R*_max_=OD340/380 in the presence of 0.1% Trixon X-100.

### Western blot analysis

Equal amounts of protein were denatured in sodium dodecyl sulfate sample buffer (2% sodium dodecyl sulfate, 62.5 mm Tris-base (pH 6.8), 10% glycerol, 5% β-mercaptoethanol and 0.005% bromophenol blue) and loaded onto a 10% sodium dodecyl sulfate-polyacrylamide gel electrophoresis gel. The separated proteins were transferred onto nitrocellulose membranes. The membranes were blocked overnight in TBS with 5% (w/v) powdered skim milk, and were stained with the recommended dilution of primary antibodies against NNAT, ATP2A1, GRP78, CHOP, p-eIF2α, CRT and β-actin. After washing, the blots were incubated with a 1:2000 dilution of goat-anti-rabbit or goat-anti-mouse immunoglobulin G antibody conjugated to horseradish peroxidase. The blots were developed with the enhanced chemiluminescence western blot detection kit (Pierce Biotechnology, Rockford, lL, USA).

### Transmission electron microscopy

Cell pellets were fixed in 2% paraformaldehyde and 2.5% glutaraldehyde in 0.1 m sodium cacodylate buffer, pH 7.4. The grids were observed on a Philips CM12 electron microscope (FEI Inc., Eindhoven, The Netherlands).

### Statistical analysis and reagents

Results are reported from at least three different experiments. Statistical analyses were performed with GraphPad Prism 5 (GraphPad Software, La Jolla, CA, USA). Comparisons were performed with the *χ*^2^ and *t*-tests. All data are reported as means±s.e.m.

Anti-NNAT antibody (catalog number: ab27266) was purchased from Abcam Inc. (Cambridge, MA, USA). ATP2A1 (catalog number: 4219S), GRP78 (catalog number: 3177S), CHOP (catalog number: 2895S), p-eIF2α (catalog number: 3398P), CRT (catalog number: 2891S) and β-actin (catalog number: 4967S) primary antibodies were purchased from Cell Signaling Technology Inc. (Danvers, MA, USA). Anti-rabbit (catalog number: 7074S) and anti-mouse (catalog number: 7076S) immunoglobulin G, horseradish peroxidase-linked antibody were also purchased from Cell Signaling Technology Inc.

PC3 cell line was purchased from American Type Culture Collection (ATCC, Manassas, VA, USA). C4-2B cell line was provided by Professor McCauley (University of Michigan, Ann Arbor, MI, USA). LNCaP cell line was obtained from Professor Peng Lee (New York UniversityNew York, NY, USA). All cell lines were identified as mycoplasma negative with PlasmoTest Kit (InvivoGen, San Diego, CA, USA). Cell lines were not authenticated recently.
