# Supplementary material for: Metformin induces ER stress-dependent apoptosis through miR-708-5p/NNAT pathway in prostate cancer
Source: Oncogenesis. 2015 Jun 15;4(6):e158–. doi: 10.1038/oncsis.2015.18 (PMC4491613; doi:10.1038/oncsis.2015.18)
Supplement: Supplementary Figure Legends [file oncsis201518x1.doc]

**Supplementary figure legends**

**Figure S1. Quantitative RT-PCR (qRT-PCR) analysis of miRNAs in prostate cancer cells. C4-2b cells were treated with 0.5 mM Metformin, 2 mM Metformin, 10 mM Metformin for 48 hours. PBS was used as a control.**

**Figure S2. miR-708-5p and metformin inhibits NNAT expression in C4-2B cells. C4-2B cells were transfected with miR-708-5p/miR-NC mimic or treated with metformin (5mM) for 48 hours. Western blot was performed to evaluate NNAT expression level.**

**Figure S3. PC3 cell line is resistant to metformin treatment.**

A) Western blot analysis of endogenous NNAT expression of PC3 and C4-2B cells. B) and C) Induction of apoptosis of PC3 cells by metformin treatment (5mM for 48 hours) was measured by Annexin Ⅴ/PI double staining and flow cytometry analysis.

**Figure S4. Metformin induces ER stress of C4-2B cells. C4-2B cells were treated with metformin at 5mM, cell lysates were extracted after 24 hours. CHOP expression level was evaluated by western blot.**
