# Supplementary figures and images for: Metformin induces ER stress-dependent apoptosis through miR-708-5p/NNAT pathway in prostate cancer
Source: Oncogenesis. 2015 Jun 15;4(6):e158–. doi: 10.1038/oncsis.2015.18 (PMC4491613; doi:10.1038/oncsis.2015.18)

Figure S1

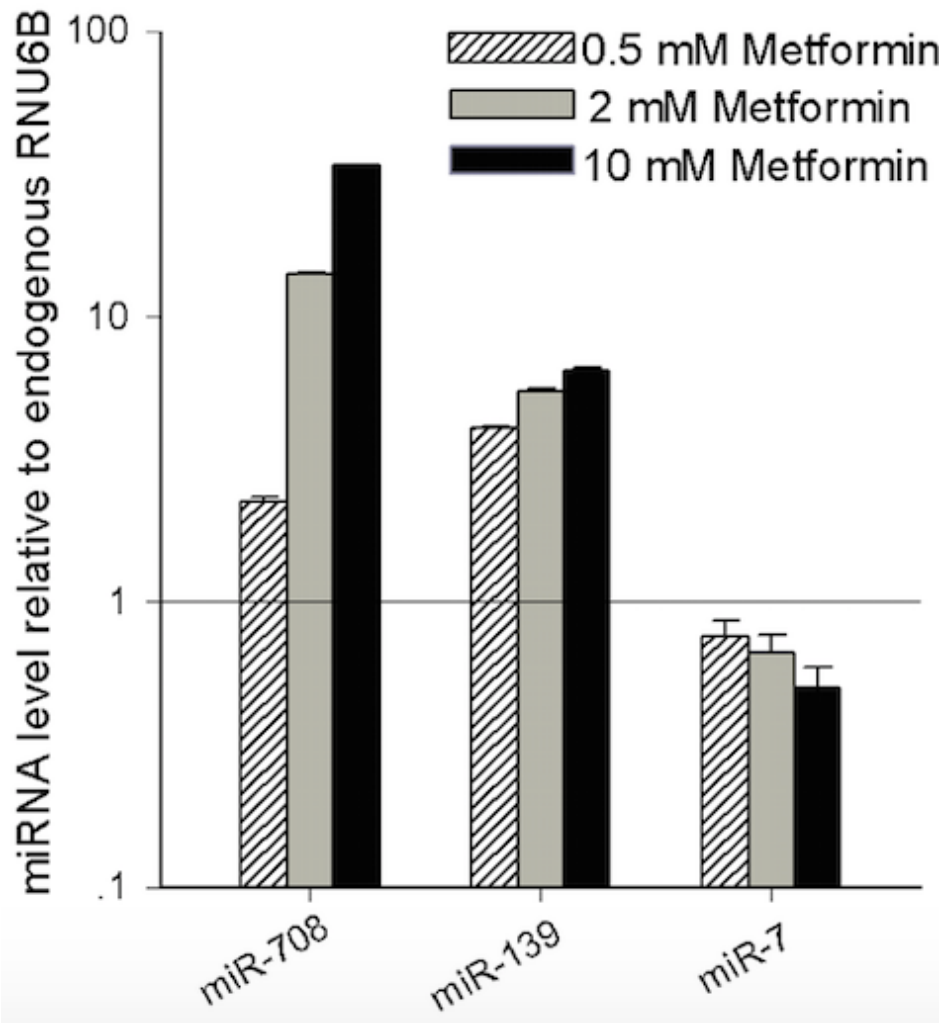

Figure S2

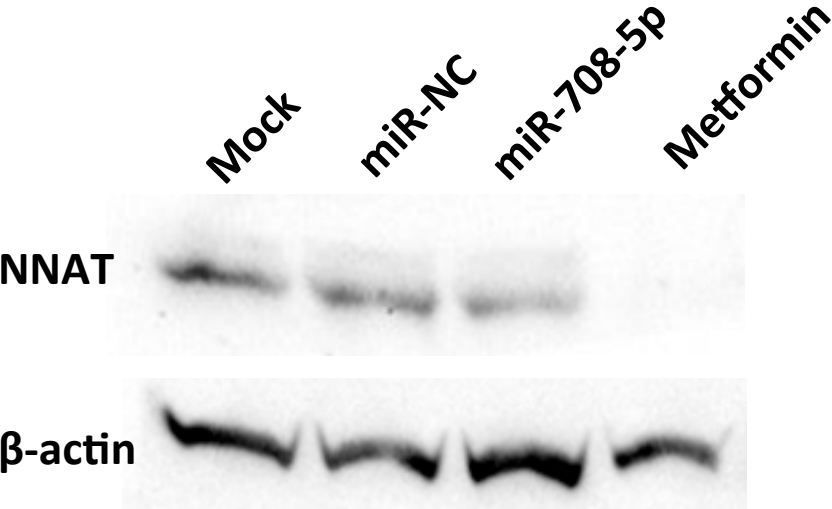

Figure S3

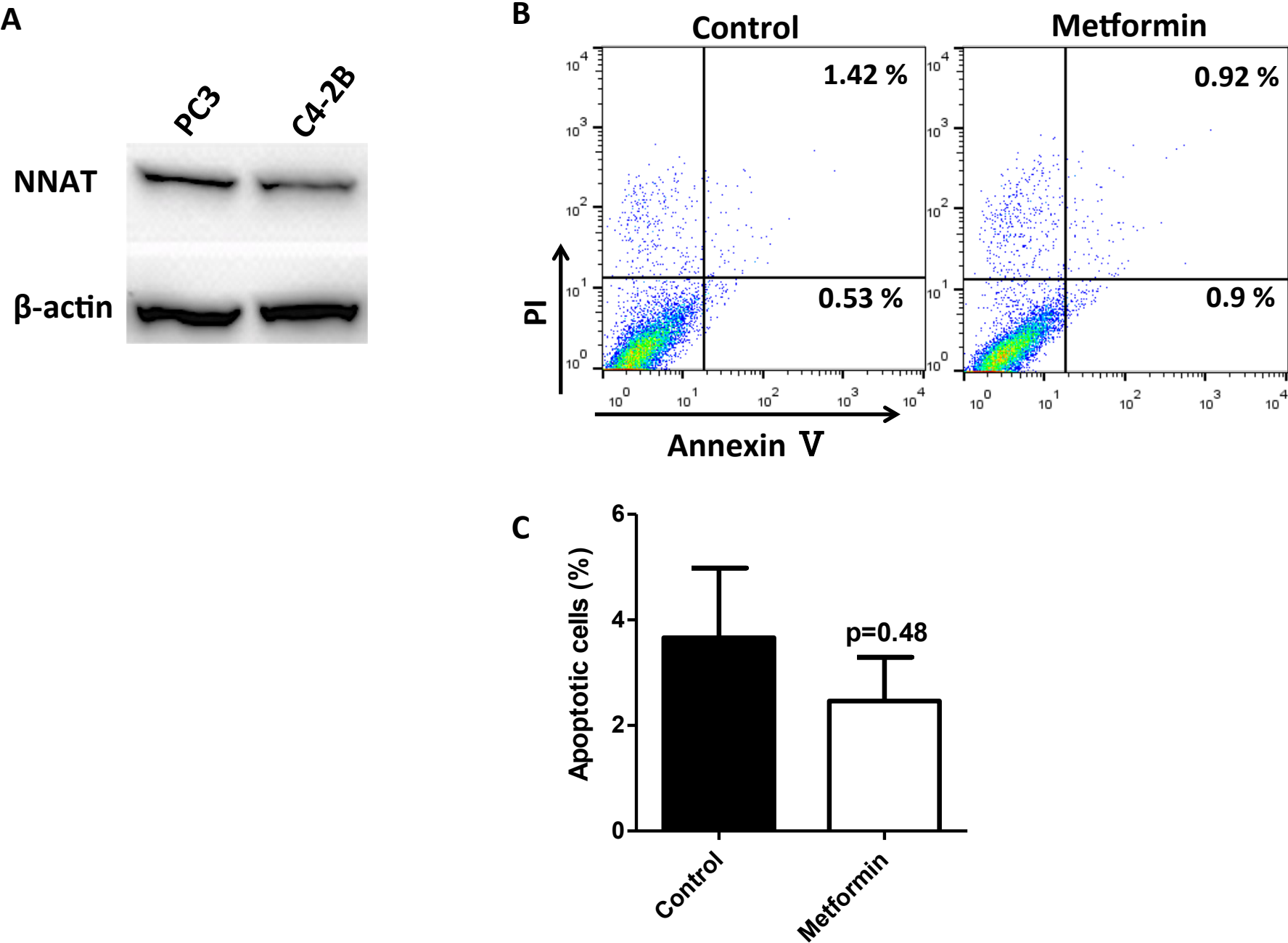

Figure S4

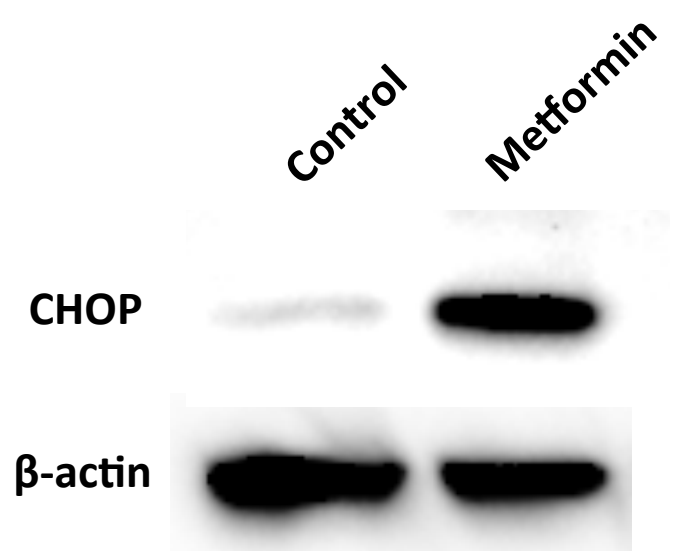

Supplement: Supplementary Figures [file oncsis201518x2.pdf]
